# Supplementary material for: Maximum entropy approach to multivariate time series randomization
Source: Sci Rep. 2020 Jun 30;10:10656. doi: 10.1038/s41598-020-67536-y (PMC7327071; doi:10.1038/s41598-020-67536-y)
Supplement: Supplementary file 1 — Supplementary information [file 41598_2020_67536_MOESM1_ESM.pdf]

Supplementary Information for
Maximum Entropy approach to multivariate time series randomization

Riccardo Marcaccioli,[1] Giacomo Livan[1,2*]

[1]Department of Computer Science, University College London, London WC1E 6EA, United Kingdom
[2]Systemic Risk Centre, London School of Economics, London WC2A 2AE, United Kingdom

[*]To whom correspondence should be addressed; E-mail: g.livan@ucl.ac.uk.

1 Explicit calculation of the partition function

We want to find a probability density function $P(W)$ on $\mathcal{W}$ such that the expectation values of a set of observables coincide with their empirical value, i.e $\langle \mathcal{O}_\ell(W) \rangle = \overline{O}_\ell$ $(\ell = 1, \dots, L)$, where $\overline{W} \in \mathcal{W}$ is the empirical set of measurements. At first, this problem may appear almost impossible to solve, given that $P(W)$ may be determined by a number of degrees of freedom way larger than the number of constraints we are imposing. However, as introduced in the main text, this can be done by using the Maximum Entropy principle, or, in other words, by adding another (functional) constraint on the probability distribution, which requires that $P(W)$ should also maximise the Gibbs entropy:

$$S(W) = -\sum_{W \in \mathcal{W}} P(W) \ln P(W) \ , \tag{1}$$

while preserving the constraints:

$$\langle \mathcal{O}_\ell(W) \rangle = \sum_{W \in \mathcal{W}} \mathcal{O}_\ell(W) \, P(W) \ = \ \mathcal{O}_\ell(\overline{W}) = \overline{O}_\ell \ , \tag{2}$$

and the normalization:

$$\sum_{W\in\mathcal{W}} P(W) \ = \ 1 \ . \tag{3}$$

Eqs. (1)-(3) define a constrained optimization problem, whose solution is found by solving the following equation:

$$\frac{\partial}{\partial P}\left[S+\alpha\left(1-\sum_{W\in\mathcal{W}}P(W)\right)+\sum_{\ell=1}^{L}\beta_\ell\left(O_\ell-\sum_{W\in\mathcal{W}}\mathcal{O}_\ell(W)\,P(W)\right)\right]=0\ , \tag{4}$$

where, as usual in such scenarios, each constraint has been coupled with a Lagrange multiplier α, β_ℓ ($\ell = 1, \dots, L$). Defining $H(W) = \sum_\ell \beta_\ell \mathcal{O}_\ell(W)$ as the Hamiltonian of the ensemble and $Z = e^{\alpha+1} = \sum_W e^{-H(W)}$ its partition function, the solution of Eq. (4) reads:

$$P(W) = \frac{e^{-H(W)}}{Z}\ . \tag{5}$$

This is the general probability density function ruling the ensemble theory we are proposing. Of course, the sum $\sum_W$ on the phase space of the system used in the above equations still needs to be properly specified.

We are going to do so while considering the Hamiltonian specified in the main paper. As pointed out above, in order to find the partition function Z of the system, we just need to sum $e^{-H(W)}$ over all possible configurations, i.e., over the set of all the $N \times T$ real valued matrices $\mathcal{W}$. Recalling the notations introduced in the main text $A^{\pm} = \Theta(\pm W)$ and $w^{\pm} = \pm W\Theta(\pm W)$, we can write the sum over the phase space as follows:

$$\sum_{W\in\mathcal{W}} \equiv \prod_{i=1}^{N}\prod_{t=1}^{T}\sum_{\substack{(0,1)\\(A^+_{it},A^-_{it})=(1,0)\\(0,0)}}\int_0^{+\infty} dw^+_{it}\int_0^{+\infty} dw^-_{it}\ . \tag{6}$$

We can now calculate the partition function Z of the ensemble:

$$
\begin{aligned}
Z = &\sum_{W\in\mathcal{W}} e^{-H(W)} = \\
=&\prod_{i=1}^{N}\prod_{t=1}^{T} \sum_{(A^+_{it},A^-_{it})=\substack{(0,1)\\(1,0)\\(0,0)}} \int_0^\infty dw^+_{it} \int_0^\infty dw^-_{it}\ e^{-\left[\left(\alpha_i^N+\alpha_t^T\right)A^+_{it}+\left(\beta_i^N+\beta_t^T\right)A^-_{it}+\left(\gamma_i^N+\gamma_t^T\right)w^+_{it}+\left(\sigma_i^N+\sigma_t^T\right)w^-_{it}\right]} \\
=&\prod_{i=1}^{N}\prod_{t=1}^{T}\left(1+\int_0^\infty dw\ e^{-\left(\alpha_i^N+\alpha_t^T\right)-\left(\gamma_i^N+\gamma_t^T\right)w} - \int_0^\infty dw\ e^{-\left(\beta_i^N+\beta_t^T\right)+\left(\sigma_i^N+\sigma_t^T\right)w}\right) \\
=&\prod_{i=1}^{N}\prod_{t=1}^{T}\left[1+\frac{e^{-(\alpha_i^N+\alpha_t^T)}}{\gamma_i^N+\gamma_t^T}+\frac{e^{-(\beta_i^N+\beta_t^T)}}{\sigma_i^N+\sigma_t^T}\right] \\
=&\prod_{i=1}^{N}\prod_{t=1}^{T}\left(1+e^{\frac{\mu^1_{it}-\epsilon_{it}}{T_{it}}}+e^{\frac{\mu^2_{it}-\epsilon_{it}}{T_{it}}}\right)\ ,
\end{aligned}
\tag{7}
$$

where all the Lagrange multipliers must be positive and we have defined the following quantities in order to make apparent the analogy with the two species fermionic gas introduced in the main text:

$$
\begin{aligned}
T_{ij} &= \frac{1}{\log\left(\sigma_i^T+\sigma_j^e\right)+\log\left(\gamma_i^T+\gamma_j^e\right)}\ , \\
\epsilon_{ij} &= \frac{1}{2}+\frac{T_{ij}}{2}\left(\alpha_i^T+\alpha_j^e+\beta_i^T+\beta_j^e\right)\ , \\
\mu^2_{ij} &= \frac{kT_{ij}}{2}\left(\alpha_i^T+\alpha_j^e-\beta_i^T-\beta_j^e-\log\frac{\sigma_i^T+\sigma_j^e}{\gamma_i^T+\gamma_j^e}\right) = -\mu^1_{ij}\ .
\end{aligned}
$$

From the above partition function, via Eq. (5) we can derive the probability density function in Eq. (5) of the main text, which quantifies the probability of drawing a specific instance W from the ensemble. The quantities defining such probability distribution have a well defined physical meaning, and read as follows:

$P^+_{it}=\frac{e^{-\left(\alpha_i^N+\alpha_t^T\right)}}{\left(\gamma_i^N+\gamma_t^T\right)Z_{it}}$ Probability of observing a positive value in the i-th time series at time t

$P^-_{it}=\frac{e^{-\left(\beta_i^N+\beta_t^T\right)}}{\left(\sigma_i^N+\sigma_t^T\right)Z_{it}}$ Probability of observing a negative value in the i-th time series at time t

$1 - P_{it}^{+} - P_{it}^{-}$ Probability of observing a missing value in the i-th time series at time t

$Q_{it}^{+}(w) = (\gamma_i^N + \gamma_t^T) e^{-(\gamma_i^N + \gamma_t^T) w}$ Probability distribution of a positive value w for the i-th time series at time t

$Q_{it}^{-}(w) = (\sigma_i^N + \sigma_t^T) e^{-(\sigma_i^N + \sigma_t^T) w}$ Probability distribution of a negative value w for the i-th time series at time t

When no data are missing, i.e. $(A_{it}^{+}, A_{it}^{-}) \neq (0,0)$, the sum defined in Eq. (6) changes and, as a result, the partition function (7) becomes :

$$Z = \prod_{i,t=1}^{N,T} Z_{it} = \prod_{i,t=1}^{N,T} \left[\frac{e^{-(\alpha_i^N + \alpha_t^T)}}{\gamma_i^N + \gamma_t^T} + \frac{1}{\sigma_i^N + \sigma_t^T} \right] \quad .$$

After noticing that $A_{it}^{+} = 0 \Rightarrow w_{it}^{+} = 0 \wedge w_{it}^{-} > 0$, the probability of drawing from the ensemble an instance W can be easily found:

$$P(W) = \prod_{i,t=1}^{N,T} \left[P_{it}^{+} \;\; Q_{it}^{+}(w_{it}^{+}) \right]^{A_{it}^{+}} \left[P_{it}^{-} \;\; Q_{it}^{-}(w_{it}^{-}) \right]^{1 - A_{it}^{+}} \;, \tag{8}$$

where the quantities in the above expression are defined as those above.

Looking at Eq. (8), we can understand how we have obtained Eq. (6) in the main text. In order to simulate a drawing of a set of time series W from the ensemble, we first need to construct a "topology" of positive events by placing a positive event in entry W_{it} with probability P_{it}^{+} and a negative event otherwise. Then we need to place a weight $W_{it} = x$ using one of the two exponential distributions $Q_{it}^{\pm}$ defined above, depending on the type of event that was assigned to W_{it}. This procedure is encompassed by the hyperexponential distribution in Eq. (6) of the main text, which can be obtained via the standard generating function approach, and whose parameters read $\lambda_{it}^{+} = (\gamma_i^N + \gamma_i^T)^{-1}$, and $\lambda_{it}^{-} = (\sigma_i^N + \sigma_i^T)^{-1}$.

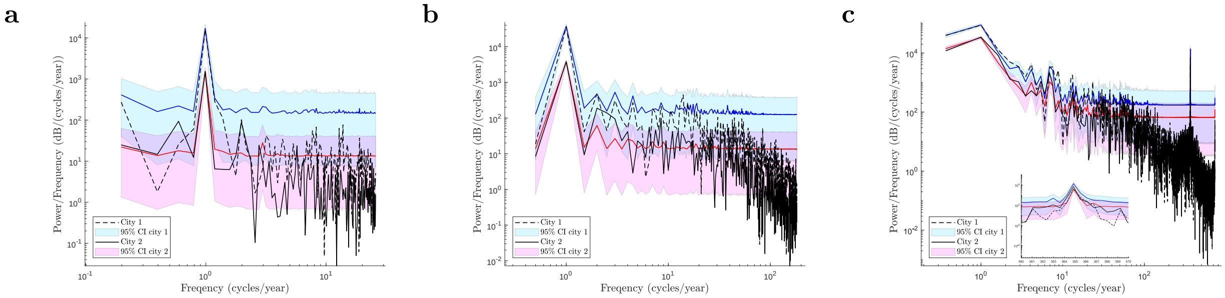

Figure S1: Ability of the esemble to preserve periodicities in the data. a) Empirical power spectrum of weekly temperatures against the average ensemble spectrum for two different cities (city 1 is Boston and city 2 is Los Angeles). **b)** Same plot for daily temperatures. **c)** Same plot for 8 hours temperatures.

2 Application to a set of temperature time series

We now apply the framework introduced in the main text to sets of time series featuring temperatures recorded at different frequencies (week/day/8 hours) in $N = 30$ different North American cities [1] (weekly data range from July 2013 to July 2018, daily data range from July 2016 to July 2018, 8 hour data range from January 2017 to July 2018). We do so in order to test the ability of our ensemble approach to capture the main features of time series whose most relevant statistical properties are markedly different from those of financial returns, which we instead studied in the main paper. In particular, our main focus will be on the ability of the ensemble to capture the periodicities that characterize temperature data at different time scales.

As done in the main text, we indicate as $\overline{W}$ the $N \times T$ data matrix (with $T = 264, 730, 2321$ in the case of temperatures recorded at the weekly, daily, and 8 hour frequency, respectively) with values rescaled to have zero mean, and we indicate as W any generic instance drawn from the corresponding ensemble. We also redefine here for convenience the matrices $A^{\pm} = \Theta(\pm W)$, $w^{\pm} = \pm W\Theta(\pm W)$. The ensemble we are going to

[1]Vancouver, Portland, San Francisco, Seattle, Los Angeles, San Diego, Las Vegas, Phoenix, Albuquerque, Denver, San Antonio, Dallas, Houston, Kansas City, Minneapolis, Saint Louis, Chicago, Nashville, Indianapolis, Atlanta, Detroit, Jacksonville, Charlotte, Miami, Pittsburgh, Toronto, Philadelphia, New York, Montreal, Boston

use is fully specified by the $6(N+T)$ constraints enforced in the following Hamiltonian (there are no missing data, which leads to $2(N+T)$ fewer constraints with respect to the general formulation outlined in the main paper):

$$H(W) = \sum_{i=1}^{N} \sum_{t=1}^{T} \left[\left(\alpha_i^N + \alpha_t^T \right) A_{it}^{+} + \left(\gamma_i^N + \gamma_t^T \right) w_{it}^{+} + \left(\sigma_i^N + \sigma_t^T \right) w_{it}^{-} \right] \ , \tag{9}$$

leading to the partition function:

$$Z = \prod_{i=1}^{N} \prod_{t=1}^{T} Z_{it} = \prod_{i=1}^{N} \prod_{t=1}^{T} \left[\frac{e^{-(\alpha_i^N + \alpha_t^T)}}{\gamma_i^N + \gamma_t^T} + \frac{1}{\sigma_i^N + \sigma_t^T} \right] \ . \tag{10}$$

In Figure S1 we show that, independently from the frequency at which temperatures are sampled, the average ensemble power spectral density captures well the relevant frequencies that characterize the empirical time series of each city. Indeed, as can be seen from panels **a** and **b**, the ensemble power spectra based on the data recorded at the weekly and daily frequency perfectly capture the six-months periodicity associated with the seasons' cycle. Panel **c** shows that the same frequency is also captured in the data recorded every 8 hours, and that when calibrating the ensemble on such data, the power spectrum also perfectly captures the daily frequency associated with the day-night cycle (see inset).

In Fig. S2 we expand the above analysis to the periodicities of moments. Panel **a** shows the empirical daily variance of temperatures recorded across the 30 cities mentioned above against the corresponding ensemble average. At first sight, the latter seems to be largely uncorrelated from the former. Yet, the corresponding power spectrum shown in panel **b** highlights that the relevant frequencies in the data (six months and one day) are captured very well, although the ensemble places additional power on such frequencies.

A somewhat similar phenomenon is shown in panels **c** and **d**, which show the daily skewness computed across all cities and its corresponding power spectra. Once again, the average ensemble spectrum places more power on the six-months and daily frequencies with respect to the empirical one. This results in a clearly discernible oscillating pattern,

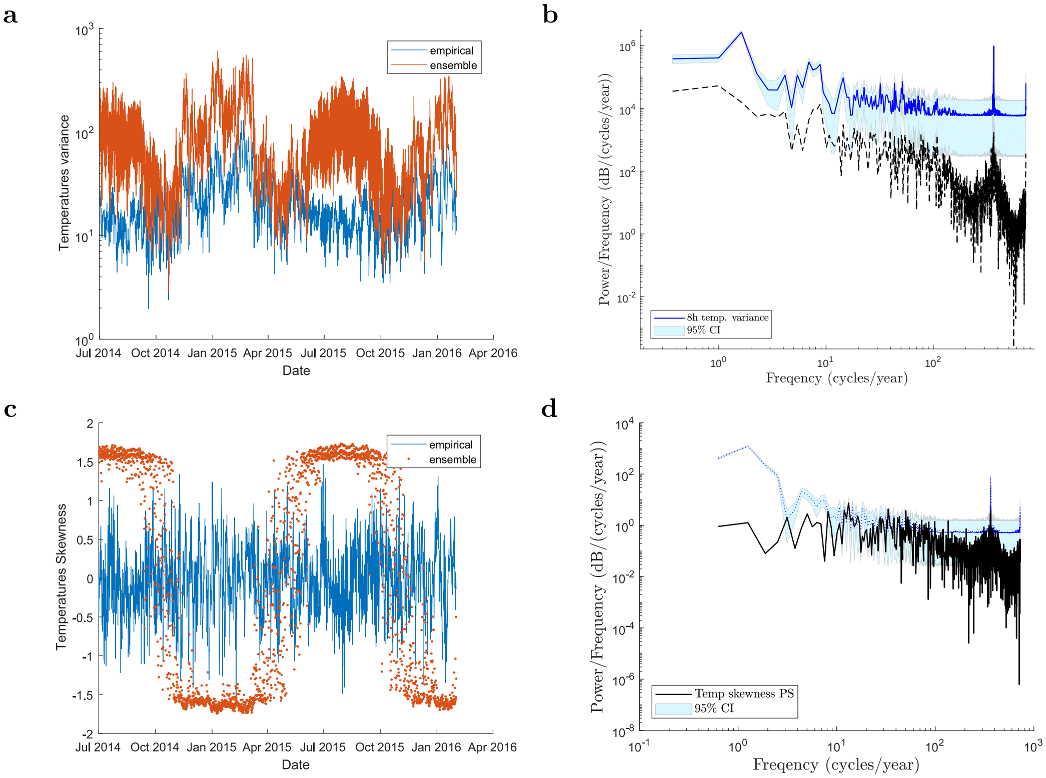

Figure S2: Ability of the ensemble to preserve periodicities in the data. a) Variance of the temperatures recorded at 8 hour intervals across all 30 cities (the blue line denotes empirical values, the orange one denotes the ensemble average). **b)** Comparison between the empirical spectrum of the 8-hours temperature variance across cities (dashed line) and the ensemble spectrum (blue line). **c)** Skewness of the temperatures recorded at 8 hour intervals across all 30 cities (the blue line denotes empirical values, the orange one denotes the ensemble average). **d)** Comparison between the empirical spectrum of the 8-hours temperature skewness across cities (dashed line) and the ensemble spectrum (blue line).

which significantly deviates from the empirical behavior. Nevertheless, these results are interesting. Indeed, as it can be seen in panel **c** positive (negative) skewness values take place during the summer (winter) months, as a reflection of higher (lower) average temperatures. Although this is a fairly trivial example, it highlights how the ensemble approach can reveal stylized trends that are genuinely informative about the dynamics of the system under study.

3 Optimal portfolio selection

Let us consider a matrix r_{it} ($i = 1, \dots, N$, $t = 1, \dots, T$) of daily financial returns, and let C be the corresponding correlation matrix (i.e., C_{ij} denotes the Pearson correlation coefficient between stocks i and j, computed over the time period $[1, T]$). The optimal portfolio problem then amounts to solving the following optimization problem for a vector $\boldsymbol{\pi} = (\pi_1, \dots, \pi_N) \in \mathbb{R}^N$:

$$\min_{\boldsymbol{\pi}} \sum_{i,j=1}^{N} C_{ij} \pi_i \pi_j \tag{11}$$

subject to

$$\sum_{i=1}^{N} \pi_i \mu_i = \mu \ ; \qquad \sum_{i=1}^{N} \pi_i = 1 \ , \tag{12}$$

where Eq. (11) expresses the minimization of the portfolio variance, while the equations in (12) express constraints on the expected returns (μ_i denotes the expected out-of-sample return of stock i, while μ denotes the portfolio's desired expected return) and on the available capital (conventionally set to one unit). The expected returns μ_i are computed – as is often customary – based on mean reversion, i.e., assuming that the return on day $t + 1$ will be minus the return on day t.

With the above positions, the solution to the optimization problem reads (see Ref. [37] in the main paper)

$$\pi_i(\mu) = \sum_{j=1}^{N} C_{ij}^{-1} (\ell(\mu) + \mu g(\mu)) \ , \tag{13}$$

where

$$\ell(\mu) = \frac{c - b\mu}{ac - b^2} \ , \qquad g(\mu) = \frac{a\mu - b}{ac - b^2} \tag{14}$$

$$a = \sum_{i,j=1}^{N} C_{ij}^{-1} \ , \qquad b = \sum_{i,j=1}^{N} C_{ij}^{-1}\mu_j \ , \qquad c = \sum_{i,j=1}^{N} C_{ij}^{-1}\mu_i\mu_j \ . \tag{15}$$

4 Sharpe ratios of portfolios in Table 3

In the Table below we report the Sharpe ratios (defined as the ration between the portfolio return and portfolio variance over a time interval) for the same portfolios considered in Table 1 of the main paper.

	P_1^{20}	P_2^{20}	P_1^{50}	P_2^{50}
$q = 2/3$	0.032<br>(-0.19 , 0.31)	0.009<br>(-0.22 , 0.23)	-0.077<br>(-0.25 , 0.15)	-0.022<br>(-0.22 , 0.28)
$q = 1/4$	-0.013<br>(-0.21 , 0.12)	-0.022<br>(-0.26, 0.23)	0.011<br>(-0.19 , 0.20)	-0.037<br>(-0.27 , 0.15)
$q = 2/3$	0.042<br>(-0.14 , 0.22)	0.041<br>(-0.16 , 0.22)	0.081<br>(-0.17 , 0.34)	0.054<br>(-0.17, 0.25)
$q = 1/4$	0.035<br>(-0.23 , 0.26)	0.035<br>(-0.17 , 0.24)	0.073<br>(-0.16 , 0.32)	0.062<br>(-0.13 , 0.34)

As it can be seen, applying the "cleaning" procedure outlined in the main paper has beneficial effects also in this case, leading to increased average Sharpe ratios and reduced uncertainty around them across board.

5 Testing for overfitting with an application on the estimation of Value-at-Risk

In this section we expand on the financial application of our ensemble approach discussed in the main paper, with a specific focus on potential overfitting issues. Namely, the number of Lagrange multipliers the ensemble depends on increase linearly with the number of constraints one wants to enforce. For instance, the multivariate case detailed in the main

paper (Eq. (7)) depends on $8(N+T)$, which, for small numbers of variables N and small sample sizes T, can be of the same order of magnitude of the number of data points $(N \times T)$ used to calibrate the ensemble. This, in turn, may raise concerns about potential overfitting issues.

We tackle the above issue by showcasing the approach's performance when using it to compute out-of-sample Value-at-Risk (VaR) estimates based on increasingly constrained (and therefore parametrized) versions of the ensemble. VaR is the most widely adopted measure of financial risk. For a given significance level α it simply refers to the $1-\alpha$ quantile of the return distribution of a certain financial stock or portfolio. The simplest procedure to estimate VaR is via historical estimation, which amounts to computing the in-sample $1-\alpha$ quantile of a financial time series of interest. However, due to non-stationarities, historical estimates are known to typically be unreliable out-of-sample, and there is a vast literature devoted to enhancing historical estimates with Monte Carlo simulations and other techniques to generate synthetic scenarios. In the following, we will apply a similar line of reasoning by computing VaR estimates as quantiles of the distributions generated by our ensemble starting from an empirical time series of interest, and we will assess their out-of-sample performance based on a number of standard statistical tests.

In the following, we consider two financial time series of length $T = 1000$ and $T = 1500$ days corresponding, respectively, to BNP returns from May 7, 2008 to March 13, 2013, and to S&P Index returns from May 7, 2008 to July 26, 2014. For each time series we proceed to compute VaR estimates with a rolling window approach. Namely, we compute an in-sample VaR estimate over a time window $[t_0, t_0+\tau]$, with $\tau = 150$ days, and assess its out of sample performance on day $t_0+\tau+1$. We do this based on the following three versions of our ensemble approach:

Model $M1$ – This corresponds to a loosely constrained ensemble based on the single time se-

ries case discussed in the paper, where we only constrain the ensemble to preserve the empirical time series' variance and the cumulative values of the data falling within each pair of adjacent quartiles (denoted as $\overline{M}_{\xi_i}$ in the main paper, with $\xi_i = 0.25, 0.5, 0.75$). Overall, these correspond to 4 constraints and Lagrange multipliers.

Model $M2$ – This corresponds to a deliberately highly parametrized model based on an adaptation of the multiple time series case. Namely, let us consider the 150 returns of interest to compute a new risk estimate and let us denote them as $r_1, \ldots, r_{150}$. We then form a 25×126 (which roughly amount to the length of a trading month and half of a trading year, respectively) matrix with such returns with the following circulant structure

$$R = \begin{pmatrix} r_{25} & r_{26} & \cdots & r_{150} & \epsilon \\ r_{24} & r_{25} & \cdots & r_{149} & r_{150} \\ \vdots & \vdots & \ddots & \vdots & \vdots \\ r_1 & r_2 & \cdots & r_{125} & r_{126} \end{pmatrix} .$$

The quantity ϵ in the upper-right entry of the matrix denotes the unknown out-of-sample return on day 151. We assume as possible values for it $\epsilon = \pm \min |r_t|$, and then generate the corresponding ensemble constraining it to preserve the cumulative positive and negative values for each row and column (denoted respectively as $\overline{S}_i^{\pm}$ and $\overline{R}_t^{\pm}$ in the main paper), which correspond to $2(25 + 126) = 302$ constraints and Lagrange multipliers[2]. We generate the ensembles for both aforementioned values of ϵ and combine the two resulting distributions for them in order to compute a VaR estimate for the return on day 151.

Model $M3$ – The same as model $M2$ with additional constraints on the number of positive and

[2]It can be shown that as long as the matrix R's sizes L_1 and L_2 are not multiple of each other, then such constraints are all linearly independent. In the case of linear dependence, the effective number of constraints decreases by at most $\max(L_1, L_2)$, which still amounts to an over-parametrized model.

negative returns recorded in each column (the equivalent of the quantity denoted as $\overline{M}_t^{\pm}$ in the main paper). In addition to the constraints mentioned above, this gives a total of $3(25 + 126) = 453$ constraints and Lagrange multipliers.

Models $M2$ and $M3$ are highly constrained (and therefore highly parametrized) ones, as they force the corresponding ensemble to preserve a very large number of local properties of the time series.

We calibrate the above models on all time windows of length $\tau = 150$ days starting on days $t_0 = 1, 2, \ldots, T - 151$ and compute out-of-sample VaR estimates for each of them, resulting in 849 estimates for BNP and 1349 estimates for the S&P Index, respectively. We then pool such estimates for both time series and assess their out-of-sample performance by means of 8 standard tests widely adopted in the financial literature. These are the traffic light, binomial, proportion of failures, time until first failure, conditional coverage, conditional coverage independence, time between failures, and time between failures independence tests (see, e.g., Nieppola, O., *Backtesting Value-at-Risk Models* for their definitions).

α	**Tests passed**		
	M_3	M_2	M_1
90%	6	4	4
95%	8	6	5
99%	8	6	6
99.99%	8	8	8

Table S1: Number of tests passed by out-of-sample VaR estimates for the BNP stock at different significance levels α.

The results – reported as the number of tests passed – are shown in Table S1 for BNP and in Table S2 for the S&P Index, for varying significance levels α. As it can be seen, the out-of-sample performance systematically improves when increasing the number of constraints, regardless of the significance level, even when pushing these to numbers

α	**Tests passed**		
	M_3	M_2	M_1
90%	5	4	4
95%	8	7	6
99%	8	7	7
99.99%	8	8	7

Table S2: Number of tests passed by out-of-sample VaR estimates for the S&P Index at different significance levels α.

close to the number of available data points. Remarkably, all tests are passed when using model $M3$ at significance 95% or higher.
